# Effects of Tai Chi on patients with moderate to severe COPD in stable phase

**DOI:** 10.1097/MD.0000000000033503

**Published:** 2022-04-07

**Authors:** Chengfang Luo, Hongjuan Jiang, Hongwen Li, Xiangyu Chi

**Affiliations:** a Department of Radiology, Shandong First Medical University, Jinan, China; b Department of Geriatric Respiratory Disease, Shandong Provincial Hospital Affiliated to Shandong First Medical University, Jinan, China.

**Keywords:** chronic obstructive pulmonary disease, exacerbation, Tai Chi, treatment

## Abstract

This study was designed to investigate the effects of Tai Chi training on moderate to severe Chronic obstructive pulmonary disease (COPD) in the stable phase. This was a 2-arm randomized clinical trial. A total of 226 COPD patients with moderate to severe in the stable phase were allocated to either the control group or the observation group. The observation of the frequency of acute exacerbation for both groups lasted for at least 52 weeks follow-up. Changes in lung function and symptom scores of health-related quality of life (St George’s Respiratory Questionnaire score) were also compared between the 2 groups. The accompanying anxiety and depressive symptoms of the patients were evaluated using the Self-Rating Depression Scale and Self-Rating Anxiety Scale prior to the procedure and 52 weeks later. Patients with moderate to severe COPD in China were divided into the Tai Chi group (n = 116) or control group (n = 110). After excluding 10 patients who fell off, 108 patients were enrolled in each group. Evidently, the matched group had higher exacerbation rate than the Tai Chi group (*P* < .05). Both groups showed no significant improvement in lung function (*P* > .05) but showed significant improvement in morbidity of acute exacerbation and quality of life (*P* < .05) compared with their former performance. Compared with regular therapy, Tai Chi also improved health-related quality of life (*P* < .05). The Self-Rating Anxiety Scale and Self-Rating Depression Scale scores of the 2 groups of patients after treatment and 52-week after treatment showed a notable decrease (*P* < .05). Overall, Tai Chi treatment was well tolerated. For moderate to severe COPD patients, regular treatment with Tai Chi can not only improve their health-related quality of life but also reduce the exacerbation rate compared with regular treatment alone. Tai Chi is recommended for COPD rehabilitation.

## 1. Introduction

Chronic obstructive pulmonary disease (COPD) is a widespread, preventable and treatable disease, characterized by persistent airflow limitation makes a significant contribution to morbidity and mortality, which causing serious social and economic burdens globally.^[1]^ It estimates that COPD will be the third leading cause of death worldwide by 2030.^[2]^ Recurrent exacerbations of COPD acutely have a stronger effect on patients, leading to a rapid decrease in pulmonary function and an exponentially higher risk of mortality.^[3]^ Based on the Global Initiative for Chronic Obstructive Lung Disease, the 2 main aims for the care of COPD patients are to improve respiratory symptoms and reduce the risk of future exacerbations. Therefore, it is critical for COPD care to implement effective measures to prevent exacerbation and alleviate disease, particularly in individuals with moderate to severe illness.^[4]^ Continual exercise intolerance and breathlessness for patients with COPD restrict their ability to carry out physical activity, leading to impairment of social and health-related quality of life.

In China, Tai Chi has been a common practice for Chinese people to exercise their minds and bodies for many centuries. Long-term Tai Chi exercises have been confirmed to contribute to the improvement of physical function, exercise capacity, and psychological state, which helps in the treatment of chronic diseases.^[5–7]^ As a good health exercise suitable for the elderly, Tai Chi has a positive impact on heart function, blood pressure, lung function, immunity, etc. It can enhance cardiopulmonary function, increase the elasticity of blood vessels, and improve the body’s self-regulation function.^[8]^ Tai Chi has been extensively used in the training modality of pulmonary rehabilitation for patients with COPD in stable phases. However, the effectiveness of exacerbation prevention remains unclear. Our study aimed to assess the efficacy and safety of new measurements that combined Tai Chi with traditional pharmacologic therapy, such as long-acting muscarinic antagonist and inhaled corticosteroid/long-acting β2-agonist in patients with COPD.

Therefore, the goal of this study was to determine the benefits of Tai Chi in preventing COPD acute exacerbations in patients with COPD.

## 2. Patients and methods

The study was approved by the local medical ethics council of Shandong Provincial Hospital and was undertaken at Shandong Provincial Hospital. All participants were recruited from outpatients of Shandong Provincial Hospital. This study was registered in the Chinese Clinical Trial Registry (http://www.chictr.org.cn) with registration number Chi-CTR-2100052564.

We used the eligibility criteria as follows: patients who were diagnosed with COPD on the basis of Global Initiative for Chronic Obstructive Lung Disease 2018 guidelines had a post-albuterol/salbutamol (FEV_1_) to forced vital capacity (FVC) ratio (FEV_1_/FVC) of <0.7 and a post-albuterol/salbutamol FEV_1_ of 30% to 79% of the predicted normal values, were aged > 40 years, had either a former or current smoking history of more than 10 pack-years, had a history of at least 2 acute exacerbations or 1 hospitalization within the previous 1 year, had stabilized clinically condition for 4 weeks preceding the study, and had signed informed consent form.

Patients were excluded if they met the following criteria: currently diagnosed with asthma, lung transplantation or pulmonary rehabilitation, hospitalization for pneumonia or COPD within 4 weeks before the visit, drug or alcohol abuse, required long-term oxygen therapy (prescribed for ≥12 h/d), antibiotic use due to lower-respiratory-tract infection within 4 weeks before visit, participated in the acute stage of a pulmonary rehabilitation project within 4 weeks before visit, and accompanied with other respiratory diseases or clinically significant medical condition including an abnormal and significant clinically electrocardiogram finding at visit determined by atrial fibrillation with a rapid ventricular rate (>120 bpm.), ventricular tachycardia or second-degree heart block Mobitz type II or third-degree heart block (unless a pacemaker or defibrillator had been inserted). Patients with severe liver or renal illness were also excluded.

### 2.1. Study treatments

All the patients provided written informed consent. The participants were randomly assigned to participate in either 52 weeks of Tai Chi combined with their usual care, or usual care alone. For balance, groups were assigned by a randomly varying blocks method. Assignments were sealed in numbering, opaque envelopes. The traditional Cheng Man-Ch’ing’s Yang-style short form of Tai Chi was used in the process. Patients of Tai Chi group were encouraged to practice Tai Chi outside at least 5 times a week for at least 30 minutes each time. They were also encouraged to practice Tai Chi outside at least 5 times a week for at least 30 minutes each time. The control group included conventional pharmacologic therapy, general exercise advice, and health education. Patients receiving routine treatment were offered Tai Chi at the end of the study. Nurses utilized telephone follow-ups once every 4 weeks to ensure the trial goes well. At the start of the trial and after 52-weeks, primary and secondary outcomes will be measured. The number of face-to-face (especially for prescriptions) is more than 2, and that of remote contacts with patients is more than 13. The use of inhaled glucocorticoids, bronchodilators (including β2-agonists, anticholinergics and theophylline), mucolytics, and antitussives was allowed to be continued during the trial. The acute exacerbation rate over 52-weeks was the main outcomes. Secondary outcomes included the time to first and second acute exacerbations, a pulmonary function test; symptom score evaluated by COPD assessment test (St George’s Respiratory Questionnaire [SGRQ]); Self-Rating Anxiety Scale (SAS) and Self-Rating Depression Scale (SDS). The SGRQ consisting of 4 dimensions (total, symptoms, activity, and impact) is a respiratory disease-specific questionnaire, and its scores range from 100 (worst) to 0 (best). SAS and SDS^[9]^ were used to evaluate the anxiety and depression degree of patients respectively. The score which is proportional to the intensity of negative emotions both ranges of 20 to 80 points. Before and after the intervention, a nurse skilled assessment was blinded to the grouping of the patients measured the score. Clinical observations and adverse events are the safety endpoints of this trial. Descriptive statistics were utilized to analyze adverse events. During the intervention, participants were asked to complete logs about adverse events, which were collected every 4 weeks. Adverse events were self-report data during phone calls. When the logs showed any adverse events that may relate to the intervention during class or home practice, such as musculoskeletal events, nurses or patients would notify study staff immediately. All outcomes testing was conducted by the research staff blinded to treatment assignment.

### 2.2. Statistical analysis

All statistical analyses were performed using the SPSS software (Version 18.0; IBM Corp., Armonk, NY). Continuous data are expressed as the mean ± standard deviation. A 2-sided *P* value of less than .05 was considered statistically significant for all outcome measures. A *t* test was used to analyze the differences between baseline and post-treatment and between-group comparisons of changes in SGRQ questionnaire ratings. The exacerbation rate of the primary end-point was analyzed using negative binomial regression analysis. For between-group comparisons of the time to first exacerbation before and after treatment, log-rank tests were performed for univariate analysis, and Cox regression analysis was utilized for multivariate analysis. The Wilcoxon rank-sum test and ANCOVA were used to compare the time interval between exacerbations following therapy across groups.

## 3. Results

A total of 226 participants with moderate to severe COPD in this study were subdivided into the Tai Chi group (n = 116) or control group (n = 110) between April 2019 and October 2021. After screening, 10 patients discontinued the study and the remaining 216 (95.6%) patients were randomized, 108 patients were included in each group, 8 patients dropped out from the Tai Chi group and 2 patients from the control group. The reasons for the dropout were unable to contact the patient, exclusion and other reasons. Demographic data, including age, sex, height, weight, BMI, and smoking history, did not differ substantially between the 2 groups (*P* = .326–.897). The number or severity of exacerbations in the previous year, baseline pulmonary function, and SGRQ score was also equivalent between the 2 groups (all *P* > .05; Table 1).

**Table 1:** Baseline characteristics in the 2 groups.

Item	Study groups		*P* value
	Tai Chi	Control	
Age, yr (mean ± SD)	68.03 ± 6.58	67.43 ± 7.34	.517
Male, n (%)	76 (70.4)	74 (68.5)	.661
Height (cm)	164.81 ± 7.22	166.13 ± 7.17	.326
Weight (kg)	60.62 ± 11.30	60.13 ± 10.69	.788
BMI (kg/m^2^)	22.09 ± 3.48	22.25 ± 3.55	.621
Smoking history, n (%)	67 (62.0)	71 (65.7)	.897
SGRQ total	61.92 ± 11.73	59.76 ± 16.81	.713
FEV_1_ (L)	1.13 ± 0.38	1.17 ± 0.31	.428
FVC (L)	2.63 ± 0.77	2.73 ± 0.79	.724
FEV_1_/FVC (%)	51.02 ± 12.03	50.45 ± 11.56	.462
Number of exacerbations in the past year	1.92 ± 0.68	1.89 ± 0.72	.723

FEV1 = forced expiratory volume in 1 second, FVC = forced vital capacity, SGRQ = St George’s Respiratory Questionnaire.

### 3.1. Acute exacerbation

**Figure 1.**
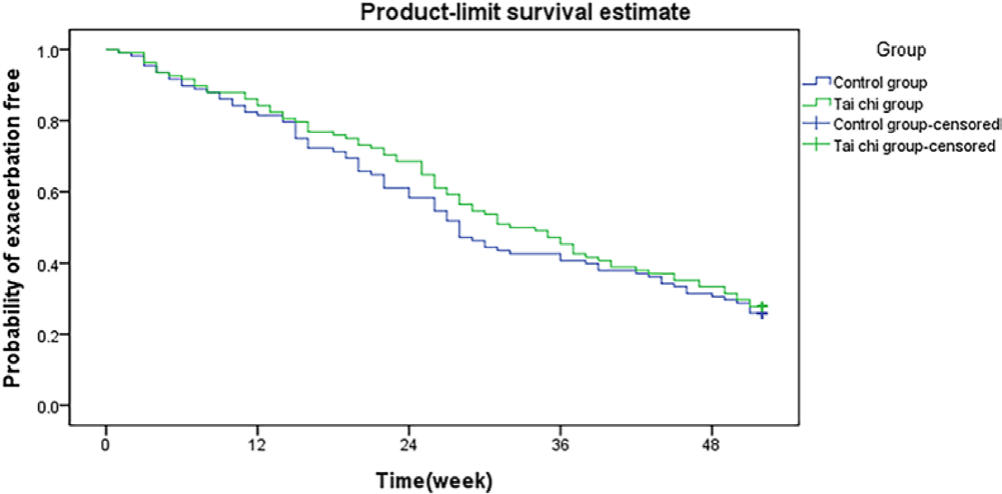
Comparison of time to first exacerbation event in patients of 2 groups.

**Figure 2.**
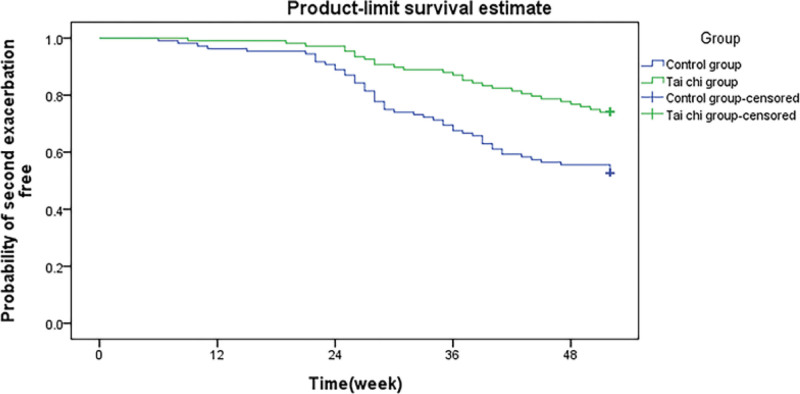
Comparison of time to second exacerbation event in patients of 2 groups.

After 52-weeks of treatment, the Tai Chi group had 132 acute exacerbations, and the control group had 205, resulting in 1.04 exacerbations per patient year with Tai Chi treatment versus 1.34 with standard care (*P* = .002). The Tai Chi group had a much lower number of exacerbations than did the control group. Exacerbation had a risk ratio of 0.586 (95% confidence interval [CI], 0.402–0.735). There was no correlation among pulmonary function or onset time. Figures 1 and 2 show the time of the first and second exacerbations, respectively. Tai Chi demonstrated a trend of delaying the start of the initial exacerbation (32.0 weeks vs 22.0 weeks, *P* = .177). The time taken for the subsequent exacerbation to occur was considerably longer in the Tai Chi group than that in the control group (HR = 0.460, 95% CI 0.301–0.687, *P* = .002).

### 3.2. Symptom score

After 52-weeks of treatment, the average change in the SGRQ-total score was −11.3 ± 14.6 in the Tai Chi group and −7.3 ± 13.1 in the control group. The between-group difference after controlling for baseline and site was −4.15 (95% CI −8.35 to −0.87), with a statistically significant difference (*P* = .022; Table 2).

**Table 2:** Comparison of SGRQ scores in the 2 groups.

		Baseline	52 wk	Diff	*P*
SGRQ, total	Tai chi	61.9 ± 11.7	49.5 ± 18.4	−11.3 ± 14.6	.032
	Control	59.8 ± 16.8	52.6 ± 17.9	−7.3 ± 13.1	.048
SGRQ, symptom	Tai chi	68.4 ± 20.6	50.6 ± 19.8	−19.2 ± 20.2	.014
	Control	71.7 ± 16.8	60.9 ± 22.2	−11.0 ± 14.3	.037
SGRQ, activity	Tai chi	81.3 ± 11.7	63.9 ± 15.9	−17.1 ± 13.6	.019
	Control	75.5 ± 19.6	65.6 ± 12.2	−9.1 ± 15.7	.066
SGRQ, impact	Tai chi	48.1 ± 16.7	36.1 ± 14.0	−13.5 ± 13.3	.023
	Control	49.8 ± 15.4	40.8 ± 18.6	−10.3 ± 16.6	.051

SGRQ = St George’s Respiratory Questionnaire.

### 3.3. Pulmonary function

Same as the control group, there was no significant improve with Tai Chi treatment for the pulmonary function as measured by FEV_1_, FVC, and FEV_1_/FVC (*P* = .858, 0.794 and 0.862; Table 3).

**Table 3:** Pulmonary function indices in the 2 groups.

Index	Visit	Group		*P* value
		Baseline	52 wk	
FEV_1_	Tai Chi	1.19 ± 0.36	1.16 ± 0.37	.528
	Control	1.20 ± 0.42	1.19 ± 0.43	.486
FVC	Tai Chi	2.51 ± 0.69	2.49 ± 0.72	.633
	Control	2.47 ± 0.71	2.47 ± 0.72	.462
FEV_1_/FVC%	Tai Chi	49.76 ± 10.95	48.99 ± 11.44	.782
	Control	50.59 ± 13.58	49.82 ± 12.94	.651

FEV1 = forced expiratory volume in 1 second, FVC = forced vital capacity.

### 3.4. SAS and SDS scores

There were no significant differences in SAS and SDS scores between the 2 groups before treatment (*P* = .669 and .751). After 52-weeks of treatment, the SAS and SDS scores of the 2 groups were much lower than before treatment, and they were much higher in the control group than in the Tai Chi group (*P* = .038 and .042). The results are shown in Table 4.

**Table 4:** SAS and SDS scores in the 2 groups.

Index	Visit	Group		*P* value
		Baseline	52 wk	
SDS	Tai Chi	58.96 ± 6.63	47.35 ± 5.78	.016
	Control	60.20 ± 7.91	52.08 ± 4.18	.027
SAS	Tai Chi	47.37 ± 5.44	38.85 ± 4.81	.013
	Control	49.12 ± 5.35	42.34 ± 4.26	.024

SAS = The self-rating anxiety scale, SDS = The self-rating depression scale.

### 3.5. Tolerability

All treatments were well-tolerated. Serious adverse events and safety concerns related to our study such as musculoskeletal events were not shown. Some patients had a short period of palpitation during the Tai Chi exercise, then they got better after having a rest. Indeed, a longer clinical follow up period is needed to ensure proper assessment of the effects of Tai Chi.

## 4. Discussion

### 4.1. Main findings

Compared with the control group, Tai Chi treatment dramatically reduced the occurrence of the acute exacerbation by 28.3 percent. Tai Chi exercise training can positively impact the immune system and particularly natural killer cells in healthy people.^[10]^ Maybe Tai Chi exercise training can interfere with natural killer cells in the blood of patients with COPD, and then impacting the acute exacerbations. We need more research to confirm these results and further clinical data on the detailed mechanism is mandated. Therefore, the overall exacerbation and exacerbation recurrence rates should be considered simultaneously to guide therapeutic treatment.

Similar to previous reports,^[11–13]^ Tai Chi, which is not beneficial to lung function, significantly improved exercise capacity over 52 weeks.

Although our study occurred during the on-going epidemic, the pandemic didn’t affect participants’ ability to engage in the intervention or to get any usual treatment. Our follow-ups were finished mainly by telephone.

Considering that prospective information on the efficacy of the usual treatment combined with Tai Chi is limited, we aimed to establish and extend the available data on patients with moderate to severe COPD. To our knowledge, this is the first study to add Tai Chi to the usual treatment, and we found that Tai Chi substantially aids in the recovery of patients with moderate to severe COPD. In addition, the Chronic Respiratory Disease Questionnaire score of the Tai Chi group was higher than that of the control group. This study also demonstrated that Tai Chi contributed to clinically meaningful improvements in the SGRQ.

Tai Chi, a traditional Chinese exercise, is not difficult to learn. Tai Chi, including soft movements and coordination of body and respiration, is favorable to the adjustments of breathing, physical activity and mind.

Anxiety and depression resemble clinical manifestations of COPD, which are sometimes difficult to identify and treat. Patients with COPD generally have a high prevalence of anxiety and depression. A previous study demonstrated that anxiety and/or depression contributes to the risk of exacerbations of COPD.^[14]^ The efficacy of antidepressant drug therapy in COPD patients with depression and anxiety is inconclusive. The improvement of physical activity limitation and social activity impairment may be the reasons for the decrease in depression and anxiety in the observed patients in both groups. Importantly, the results suggest that Tai Chi can reduce both anxiety and depression more effectively than routine treatment. Tai Chi emphasizes the combination of physical and breathing activities, the exerciser can enhance breathing muscle strength, which can increase the body’s intake of oxygen, which plays an important role in guiding the improvement of anxiety and depression symptoms. Regardless of the fact that psychological therapy, pulmonary rehabilitation, and collaborative care models can relieve the depression and anxiety symptoms of COPD patients, the findings have not been supported by evidence from long-term follow up. Therefore, more randomized controlled trial research is needed.

Tai Chi is a low to moderate intensity exercise, which is mainly marked by concentration, structural adjustment and flexibility to relax the body and mind. It is easy to learn and does not require much effort, therefore, practically anyone may practice it at home. As a result, this study could be useful in identifying the benefits of Tai Chi on quality of life and psychology.

Comparing to the usual short-term pulmonary rehabilitation project for COPD patients who have completed, Tai Chi could be a long-term and minimal budget to maintain physical activity. It is necessary for patients with COPD to try various lifestyle behaviors, especially those that can positively influence the disease course. Consistent with previous studies, most COPD patients thought that Tai Chi was a joyful and interesting program to promote exercise self-efficacy, and they intended to continue Tai Chi exercise after the study.^[15]^

One of the fundamental limitations of this study was that the participants could not be blinded owing to the nature of the intervention. Because the experimental group was required to practice Tai Chi, the participants were in a position to tell whether they were in the experimental or comparator group. This information may have an effect on the study’s findings by affecting other behaviors that may differ between the groups.

Large-scale randomized controlled trials are necessary because of our small number observers.

## 5. Conclusions

In conclusion, compared with the usual treatment for the moderate to severe COPD patients who received, Tai Chi added to the customary treatment could improve mobility. More importantly, no additional safety concerns were identified for this different treatment. Therefore, Tai Chi is recommended for rehabilitation of the patients with COPD. However, additional larger-scale randomized controlled trials that can provide high-quality evidence are still necessary to verify the long-term effect of Tai Chi, helping to estimate targeted measurement of rehabilitation interventions for patients with COPD.

## Author contributions

**Data curation:** Hongjuan Jiang.

Formal analysis: Xiangyu Chi.

Funding acquisition: Xiangyu Chi.

Methodology: Hongwen Li.

Writing – original draft: Chengfang Luo.

Writing – review & editing: Xiangyu Chi.
